# Drinking on the wing: water collection in polarotactic horseflies

**DOI:** 10.1007/s00359-023-01657-3

**Published:** 2023-07-21

**Authors:** Uroš Cerkvenik, Gregor Belušič

**Affiliations:** Department of Biology, Biotechnical Faculty, University of Ljubljana, Večna Pot 111, 1000 Ljubljana, Slovenia

**Keywords:** Polarotaxis, Water drinking, High-speed videography, Horseflies, Polarisation vision

## Abstract

Many insects detect water bodies by observing the linearly polarised light which is reflected from the water surface. Polarotactic horseflies exhibit acrobatic manoeuvres above the water and are able to plunge on its surface, collect a droplet and fly away. This behaviour is extremely fast and has not yet been analysed. We recorded the flight patterns and kinematics of drinking horseflies using a pair of high-speed cameras. The animals of both sexes are attracted to water puddles where they make short, millisecond pitstops to collect a droplet of water that is then presumably drank “on the wing”. Before the collection, the flies perform several low-altitude flybys above the puddle. After a few passes, the fly suddenly reverses its body orientation, decelerates, briefly touches the water surface and immediately flies away, usually with a droplet carried between its front legs. During the approach flight, the horseflies fly low but do not show any angular preference. Thus, they view the reflections from the sky, sun, or vegetation with a wide band of ventral ommatidia. Polarotaxis in drinking horseflies is a very robust visually guided behaviour, which operates at a broad range of intensities and various spectral compositions of reflected light.

**Supplementary Information:**

The online version contains supplementary material available at 10.1007/s00359-023-01657-3.

## Introduction

Water is a very important visual cue for many animals, but visual recognition of water bodies is not a trivial behavioural task. Water bodies have a characteristic smooth or wavy surface, usually a horizontal alignment, and a complex, highly variable colour. The colour depends on suspended particles, reflections from the bottom, spectral filtering by the water molecules, and surface reflections of objects such as sky or vegetation (Horváth and Kriska 2008). For animals with highly resolving, camera-type eyes, these features can be exploited for the visual identification of water; however, they may be irrelevant to animals with lower-resolution vision. For instance, a body of water such as a puddle, viewed through insect compound eyes with typically 1° spatial resolution, may occupy the visual space of merely a few ommatidia, which is insufficient to resolve a flat reflective surface. Thus, insects seeking or avoiding water must rely on other visual cues.

Water surfaces reflect horizontally polarised light (HPL), whereas the surrounding objects such as foliage or soil reflect unpolarised light or a fragmented light pattern, polarised at many different angles (Horváth 1995). Insect photoreceptors are intrinsically sensitive to polarised light (Snyder and Laughlin 1975); thus, HPL is potentially a salient visual cue that guides polarotaxis, attraction to polarised light. Polarotactic insects are those that depend on water for reproduction, feeding, drinking or orientation, such as dragonflies, long-legged flies, mayflies, midges, water striders or horseflies (Kriska et al. 1998; Bernáth et al. 2008; Horváth et al. 2011; Egri et al. 2012; Farkas et al. 2016; Obayashi et al. 2021). Reflections from water are highly horizontally polarised only when viewed from around the Brewster’s angle, i.e. around 55° from the normal. These reflections are mixed with the unpolarised light, reflected from the bottom or the water column, which reduces the overall degree of polarisation (DoP) of water-reflected light (Horváth 1995). Polarotactic insects evolved highly polarisation-sensitive (PS) photoreceptors for object detection that are often localised in specialised regions in the ventral retina (Wernet et al. 2015; Heinloth et al. 2018). A typical example of compound eyes with a specialised zone is found in the common backswimmer *Notonecta glauca* (Schwind 1983, 1991). In this species, the photoreceptors with high PS occur in a band across the ventral retina, where the optical axes of the ommatidia are aligned with the Brewster’s angle during approach to water.

**Fig. 1:**
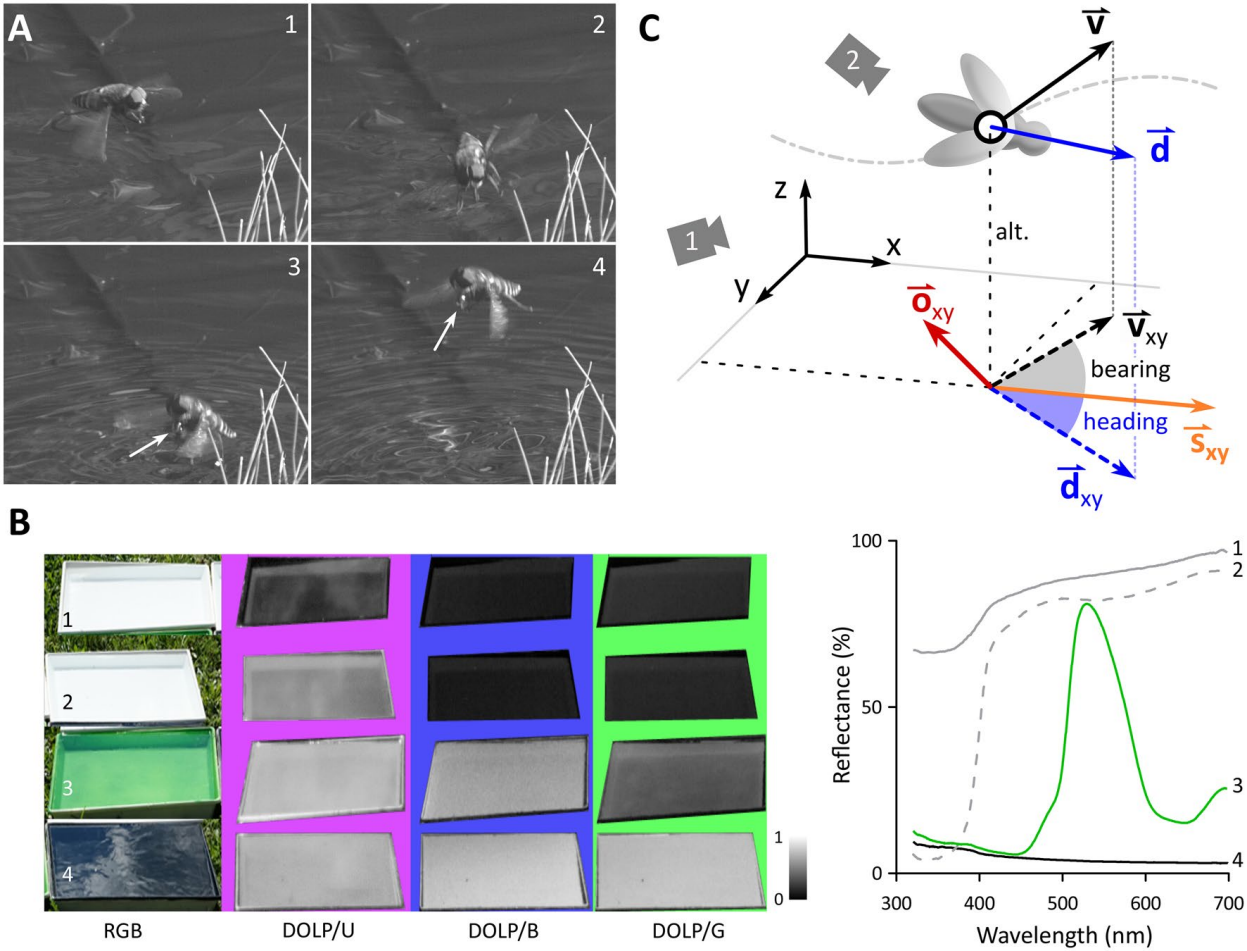
Drinking behaviour, water trays and recording setup.** A** Frames from a video showing detailed horsefly drinking on the wing manoeuvre with approach (1), sharp turn before landing (2), take-off (3), and departure (4) with a droplet in between its front legs (white arrows). **B** Photos of differently coloured water trays using a regular camera (RGB; Sony RX10-III) and one capable of detecting the degree of linear polarisation (DOLP) fitted with a UV (U), blue (B), or green (G) filter. The photographs were taken at the viewing angle where the DOLP was maximal. This angle was the same for all images and trays. The reflectance of empty trays is shown in the graph on the right. The DOLP is inversely proportional to the intensity of reflection from the bottom of the tray. **C** Schematics of the experimental setup with quantities extracted from the 3D reconstructions. *alt.* altitude above the water surface, ***v*** velocity, ***d*** animal’s body direction, ***v***_*xy*_* and ****d***_*xy*_ projections of the velocity and body direction vectors onto the XY plane, ***s***_*xy*_ sun direction (azimuth), ***o***_*xy*_ observer direction. Heading and bearing were defined as angles between the sun azimuth and, respectively, the **d**_xy_ and the **v**_xy_

Horseflies exhibit polarotactic behaviour and seek water for mating and oviposition (Horváth et al. 2008). In fact, horseflies are attracted by any surface on the ground, reflecting HPL (Egri et al. 2012). In females, the high PS receptors are spread semi-stochastically across the entire retina, with higher densities in the ventral part of their compound eye (Meglič et al. 2019); the males have a dorsal area specialised for the detecting females, while the ventral retina is similar to females (article in preparation). Horseflies were described to briefly touch the water during flight (Horváth et al. 2008). Our observations of this behaviour revealed that the horseflies actually plunge into water to collect droplets for drinking (Fig. 1A; Mov. 1). Horsefly flight pattern is similar to other fly species and consists of straight segments separated by rapid turns termed body saccades (Collett and Land 1975; Tammero and Dickinson 2002; Muijres et al. 2015). A particularly prominent body saccade is observed during water collection when the animal rotates by approximately 180° in the horizontal plane (yaw), decelerates and drops on the water surface (Mov. 1–3). This results in characteristic flight patterns around water puddles that seem to be influenced by the HPL.

HPL reflections from water are an unstable visual cue (Stockhammer 1959; Waterman 1981). Their intensity varies across orders of magnitude, as the reflected sunlight is much more intense than the light, reflected from the sky or terrestrial objects, whereas their spectral composition depends on the spectral properties of reflected objects and water in the column. Additionally, the vertically polarised skylight that occurs in the north and south during sunset and sunrise is not reflected from the water surface under Brewster’s angle and appears as a dark patch (Gál et al. 2001). Thus, we hypothesised that the horseflies approach water under specific angles and altitudes, where the HPL reflections are most intense and stable. To understand the attraction to a highly complex visual cue in detail, we analysed the horsefly approaches to water using high-speed 3D videography.

## Materials and methods

### Animals

The horseflies (*Tabanus bromius*) were filmed on four afternoons in August 2021 between 13:30 and 17:00 (cloud cover: none or partly cloudy, temperature: 25–35 ℃) in a meadow surrounded by forest (45°56′35.1ʺN 14°26′56.5ʺE). We recorded 73 events where the horseflies touched the water but discarded seven events where the flies either only skimmed the water surface or flew uncontrollably and crashed into the tray’s edge. Each water-landing/drinking event was assumed to be of a different animal.

### Attraction preferences

A suitable attraction stimulus was found by presenting the horseflies with four differently coloured water trays (black, green, UV-absorbing white, UV-reflecting white) placed in the meadow (Fig. 1B) and counting the number of visits within a 5-min time window. The colours were chosen based on the characterisation of the horsefly retina, which possesses polarisation and colour-sensing photoreceptors with peak sensitivities in the UV, blue and green range (Meglič et al. 2019). The trays reflected light with different spectral composition, intensity and degree of polarisation (DOLP), which was measured with a monochrome polarimetric camera (Phoenix, Lucid Vision labs, Germany), fitted with a quartz lens (FL-BC2528-VGUV, Ricoh, Japan) and bandpass filters (40 nm bandwidth, centre wavelengths 360, 450, 525 nm; Edmund Optics, UK). The white trays reflected across most of the spectrum which resulted in low overall DOLP of reflected light (Fig. 1B). The DOLP across all regions of the spectrum was the highest in the black-coloured tray (Fig. 1B).

### Recording setup

The setup consisted of either a dark red or a black circular water tray (radius: 17 cm, height: 5 cm) placed on top of a dark (green or black) tarpaulin that was spread on a gravel road. The tarpaulin increased the contrast between the animal and the background, which facilitated video digitisation. The tray was filmed from two different angles using two consumer grade Sony RX10-III cameras (pixel resolution 1920 × 1080) mounted on tripods positioned between 0.8 and 1.20 m from the tray and operating at a sampling frequency *f*_s_ = 250 fps, which allowed 4 s long flight recordings. The cameras were triggered remotely using a cable system which allowed the experimenter to be stationed approximately 2 m from the tray. The camera and observer positions were recorded in the lab journal and changed randomly during and across experimental days to account for their potential influence on the animal behaviour (SI Fig. 6). The aperture, focus and magnification were set manually for each camera position and varied during and across recording days. The setup and analysis schematics are shown in Fig. 1C. The videos and data were analysed in Python (3.8), except where stated otherwise.

### Video synchronisation

The Sony RX10-III cameras cannot be synchronised with currently available commercial hardware. Instead, the recorded videos were synchronised during post-processing. This was done using a custom-built LED counter consisting of an Arduino Mega 2560 microcontroller running a reflected binary code (i.e. Grey code) operated at 25 Hz. The microcontroller was connected to two sets of eight LEDs that were pointed towards the different cameras. During post-processing, the LED intensity pattern was first extracted using ImageJ (1.53 t). These intensities were converted into the reflected binary code and then into integers using a custom Python code. Finally, the frames from the two cameras were aligned in time using cross-correlation with the accuracy of a single frame.

### Camera calibration and 3D reconstruction

Calibration was performed using a DLT method and a checkerboard pattern of known size, which was filmed at different orientations in both camera views. The DLT coefficients were calculated using the StereoMorph package (1.6.7; (Olsen and Westneat 2015)) in R (4.2.1), which was also used for reconstructing 3D flight trajectories from the two camera views. The checkerboard reconstruction error was generally low, in subpixel range along the epipolar lines and in the range of 10 pixels towards the edge of the camera views. Calibration was performed every time we changed the camera position or settings.

### Digitisation

To obtain the animal orientation during flight, we digitised the front and rear of the fly bodies in synchronised videos. Initial digitisation was done in DeepLabCut (DLC; (Mathis et al. 2018; Nath et al. 2019)) running on Google Colab servers. The DLC model was trained on a subset of 11 drinking events before analysing other videos. Care was taken that the training set covered a wide range of flight paths/directions and all camera views and lighting conditions experienced during recordings. The DLC digitisation was prone to errors, particularly during water landing and at low animal-background contrast. We amended the DLC errors with a second, manual digitisation using a custom Python code.

The solar azimuth was obtained by manually digitising the shadow of a vertical beam in one recording event per calibration. The beam was removed before taking further recordings as not to obstruct the view of the animal and its flying behaviour. The shadow direction during drinking events with no beam present was extrapolated based on the time of the recording, the GPS location of recording setup and meteorological data available online.

### Trajectory alignment and filtering

The 3D trajectories from different calibrations were reconstructed in arbitrary world coordinate systems and needed to be aligned in space and time. Time alignment was done manually, by assigning the frame in which the animal first touched the water to time *t* = 0 s. Alignment in space was done programmatically such that in all the reconstructions, the horizontal plane vector (i.e. the plane of the water surface **n**) pointed along positive *Z* axis, the sun azimuth pointed along the positive *X* axis, and the location of the water contact was in the origin of the world coordinate system. This was for each reconstruction achieved by two rotations and one translation. First, the horizontal (*XY*) plane was found by fitting a plane through at least four digitised landmarks on the ground or on the water tray. All points were rotated such that the plane normal (**n**) that pointed towards the flight path aligned with the positive *Z* axis. Second, we calculated the projection and rejection vectors of the shadow vector onto the *XY* plane defined by **n** and rotated all points such that the shadow projection vector aligned with the negative *X* axis (i.e. rotation along the *Z* axis). The shadow vector and its *XY* projection differed in the order of 1°, which indicates a low reconstruction error of our dataset. Finally, all points were 3D translated by subtracting from them the coordinates of the point in the [−20 ms, 20 ms] time range with the lowest *Z* coordinate.

**Fig. 2:**
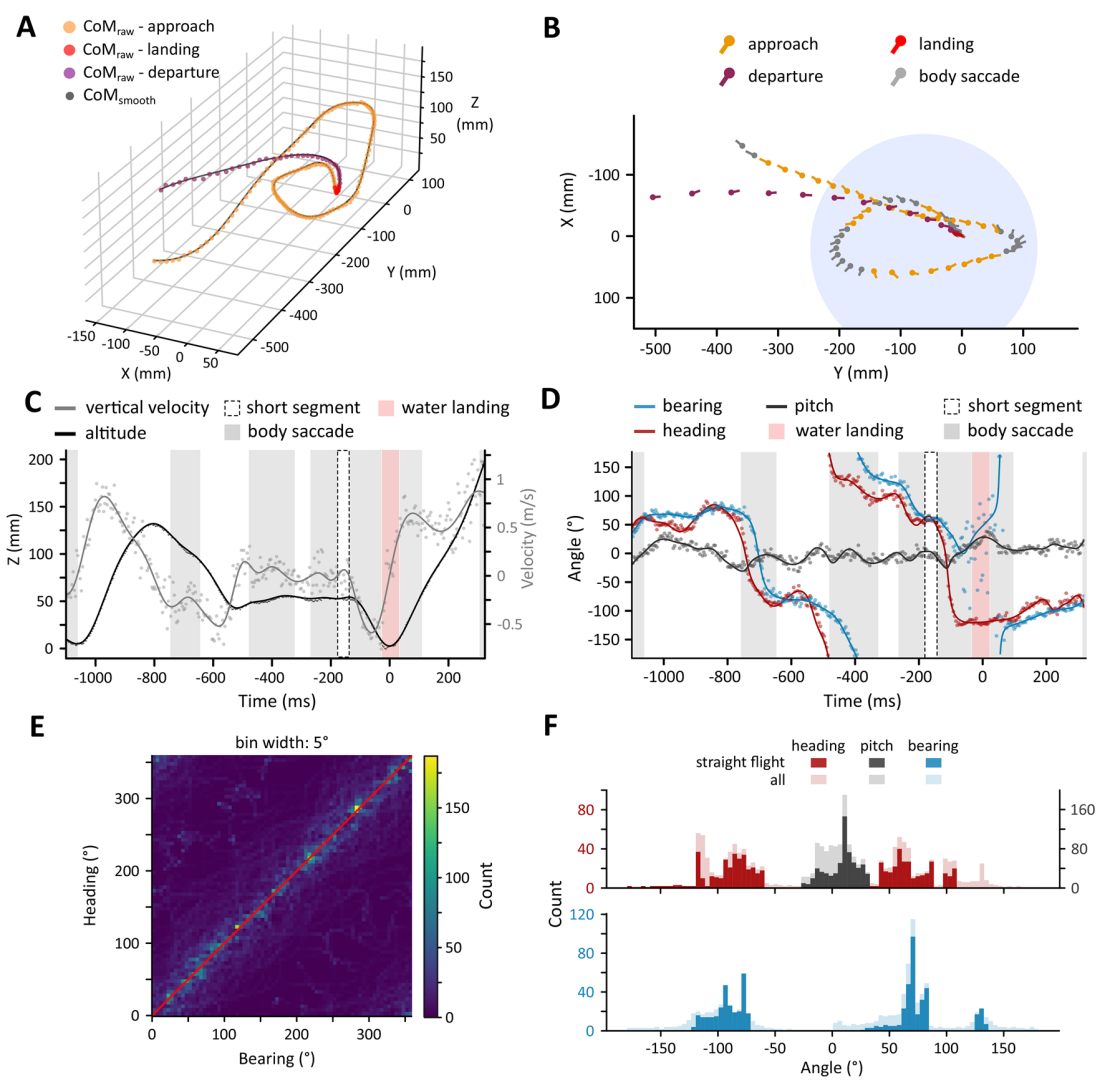
Flight path analysis example.** A** 3D trajectory of centre of mass (CoM) divided into three stages: approach (orange), water landing (red), and departure (purple). Black line indicates the up-sampled and filtered 3D CoM trajectory. **B** 2D projection of the 3D path in (**A**) showing the digitised animal (full circles indicate the head) and the location of the water tray (blue circle). Colour scheme same as in (**A**), with the addition of grey body saccades isolated using the 2D CoM curvature. The body saccades were omitted from directional analysis. **C** Flight altitude (black) and vertical velocity (grey) in time. Dots: raw data, lines: filtered and up-sampled data, grey rectangles: body saccades, red rectangle: landing phase, dashed rectangle: period of steady flight that was excluded from the analysis because of its short duration (<10 ms). **D** Bearing (blue), heading (red), and pitch (black) during the flight shown in (**A**) and (**B**). The change in heading occurs first, followed by the change in bearing (shift along the time axis of the blue line compared to the red). The changes in heading and bearing are similar during approach and departure but differ just before and during the water landing. The animal turns by approximately 180° (heading) while retaining its bearing, which changes during the take-off. Pitch oscillates around 0° during normal flight, except during descent, when the horsefly distinctly pitches up, thus contacting the water surface abdomen-first. Dots: raw data, lines: filtered and up-sampled data, grey, red, and dashed rectangles same as in (**C**). **E** A heatmap of the heading and bearing angles of all recorded flights. Despite the slight time lag between the two, the change in bearing closely follows the change in heading. Red line indicates the trend if bearing would always equal heading. **F** Heading (red), bearing (blue), and pitch (black) histograms of the entire flight (light colours) and during periods of steady flight (solid colours). Excluding the body saccades from the analysis reduces the noise in directional data

The aligned 3D trajectories of the front and the rear were filtered using a second-order low-pass Butterworth filter (cut-off frequency: 100 Hz) and then up-sampled by a factor *F*_up_ = 3 (Fig. 2A). This significantly reduced the noise in the reconstructions, slightly reduced the curvature of the flight path, but retained most of the flight details (Fig. 2A). The centre of mass (CoM) was approximated by taking the mean of the front and rear 3D coordinates.

### Wind speed

Wind characteristics during the fieldwork were not quantified, but instead noted down qualitatively. The experiments were done during the summer in a meadow surrounded by hills covered with dense forests and we experienced little to no wind. An unofficial archive of meteorological data curated by a meteorological association Zevs can be found online (http://rakitna.zevs.si/weather27/index.php). The average recorded wind speed for the village Rakitna, which is situated a couple of kilometres from the test site, was low (2.65 km/h) and was likely less on our test site due to the vicinity of the forests. Considering the flight capabilities of horseflies, we conclude that the wind had no or only minor effects on their flight behaviour.

### Flight analysis

**Fig. 3:**
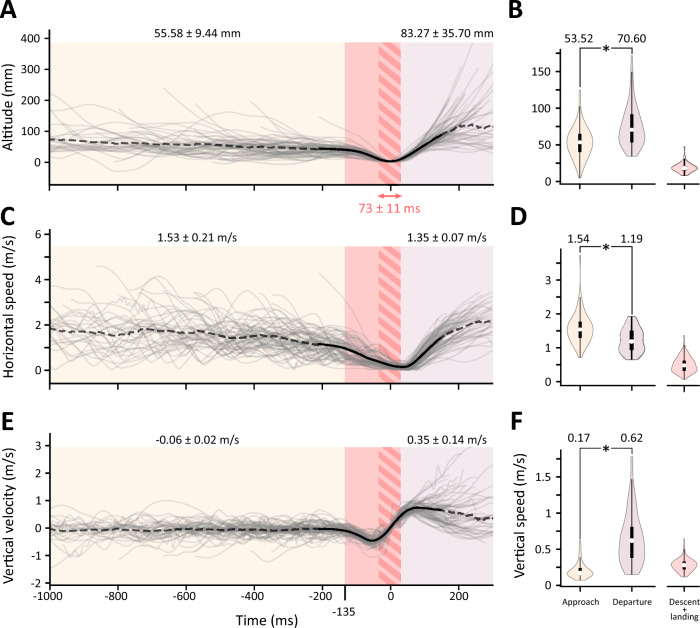
Water tray inspection is done at slow speeds and low altitude. All analysed flights aligned in time (left) with associated violin plots of the different flight phases (right). The moment of first water contact is set to 0 ms. Approach phase (orange), water collection phase (red), departure phase (purple). Red hatching indicates the “strict” water-landing phase (altitude <10 mm). Mean time (± standard deviation) spent on water surface was 73 ± 11 ms. **A**, **B** The horseflies preferentially inspected the water at low altitudes. **C**, **D** Horizontal speed during drinking on the wing is generally low and slightly higher during approach compared to departure. During water landing, the animals retain some of their horizontal speed. **E**, **F** The low negative vertical velocity during inspection indicates a gradual decrease in altitude during inspection phase of the flight. The fast descent towards the water surface typically started around *t* = −135 ms before water contact (descent onset threshold was taken as 20% of peak descent velocity). **A**, **C**, **E** Grey lines: individual sequences; dashed black lines: mean values; full black lines, length of the shortest analysed flight; mean values (± standard deviation) in different sections written above the respective sections (**B**, **D**, **F**). Violin plots show the distribution of individual flight means per phase. White circles: distribution medians, black box and whiskers plots: quartiles. Asterisks indicate statistically significant differences between approach and departure phases (Table 1)

We divided the individual flights into three phases: approach, water collection/landing, and departure. The water collection/landing was slightly different when analysing the directionality or when analysing the flight kinematics (altitude, speed). In directional analysis, the water landing was defined as the time window around *t* = 0 s where the altitude of the CoM was <10 mm, which is roughly half the body length of a horsefly (Fig. 2A–C**)**. In flight analysis, the water collection/landing phase also included the descent to the water surface, i.e. the typical flight manoeuvre the horseflies perform just before landing on the water (Mov. 1, 3). The descent onset was determined as the moment when the mean vertical velocity reached 20% of its peak negative value (−0.09 ms^−1^) which on average occurred at *t* = −135 ms (Fig. 3). Everything occurring before the water collection/landing was considered the approach, while everything occurring after was considered the departure.

### Flight speed and altitude

The horizontal speed was calculated as the magnitude of the CoM velocity vector projected onto the *XY* plane. The vertical velocity was obtained by taking the derivative of the CoM *Z* coordinate with respect to time (d*t* = 1 / (*f*_s_ · *F*_up_)), while the vertical speed was taken as the absolute value of vertical velocity. The flight altitude was taken as the CoM *Z* value.

In time-aligned sequences (Fig. 3A, C, E), we calculated the mean values of altitude, horizontal speed and vertical speed at each time point (dashed and solid black lines) and used the obtained curves to calculate the mean values in each flight phase. However, for statistical analysis, we first calculated the mean values per animal in its approach and departure phases. The two (coupled and skewed) distributions of means were then characterised by calculating their medians and quartiles (Fig. 3B, D, F) and finally compared using Wilcoxon signed-rank test. The effect of calculating the averages on the data distribution was checked by comparing the distributions of averaged data with those of non-averaged data, and with means obtained from segmented flight paths used in directional analysis (SI Fig. 4).

### Body orientation and flight direction

The horsefly flight pattern consists of straight segments separated by rapid body saccades (Fig. 2B). This strategy is hypothesised to reduce motion blur (Schilstra 1999; Land 1999) and we, therefore, focused our analysis on the segments of straight flight. Furthermore, we limited our analysis to rotations around side-to-side axis (pitch) and vertical axis (yaw). The latter was done by projecting the 3D trajectories onto the *XY* plane (i.e. setting the *Z* coordinates to 0). We defined straight flight segments as segments of 2D CoM trajectory with the radius of curvature (*r*_κ_ > 210 mm; Fig. 2B). This value was chosen because it is much larger than the body length of the animals (approximately 20–25 mm) and provided consistent segmentations across the entire dataset. The segmentation quality was assessed by visual inspection of the data and by comparing the data distributions of raw data, phase means and segment means (SI Figure 4). Additionally, we performed a sensitivity analysis using two other thresholds, namely *r*_κ_ > 105 mm and *r*_κ_ > 320 mm, which had little effect on our directional analysis (SI Table 1). During flight, two horizontal directions can be distinguished: the direction in which the animal is moving (i.e. its bearing) and the direction in which the animal is looking/pointing (i.e. its heading). As shown in Fig. 1C, we defined both directions with respect to the sun azimuth. Bearing was defined as the angle between the animal’s CoM horizontal velocity vector and the sun azimuth. The CoM velocity was calculated as the time derivative of the change in CoM *XY* coordinates (Fig. 1C**)**. The heading direction was defined as the angle between the animal’s horizontal body orientation (i.e. the vector pointing from its rear to its head) and the sun azimuth (Fig. 1C).

The bearing was more stable than the heading (Fig. 2D) and was used to analyse the animal’s directional preferences during flight. However, as mentioned above, the bearing and heading direction are different and not necessarily in sync during flight. Generally, the change in bearing lagged slightly behind the change in heading (time shift in Fig. 2D). Additionally, the two quantities differed greatly during the water-landing manoeuvre, where the fly quickly changed its heading for approximately 180° while retaining its bearing, which changed at the time of the take-off (Fig. 2D). Nevertheless, when comparing the bearing and heading angles in our entire dataset, the overall changes in bearing accurately reflected those of the heading (Fig. 2E; SI Figure 1A). Additionally, bearing and heading were not affected by the flight altitude (SI Figure 1B). Considering all of the above, we used the bearing angles during approach and departure, and heading angles during the water-landing phase in our directional analysis.

### Directional analysis

The isolation of straight flight segments from body saccades reduced the noise in directional data. Angles that occur less often during flight (i.e. during turns) were omitted, while the directions of steady flight were retained (Fig. 2F). For each isolated flight segment, we calculated both its mean direction angle (circular mean of instantaneous directions) and length (*r*, vector sum of the instantaneous direction vectors). We calculated mean bearing vectors for approach and departure segments, and mean heading vectors for the landing phase. The obtained vector distributions were analysed using the Moore’s modified Rayleigh (MMR) test (Moore 1980; Dreyer et al. 2021) using vector lengths as weights for each of the flight phases. The chosen weights increased the contribution of long flight segments with little change in bearing/heading (i.e. highly directed segments). The MMR test assumes unimodal data distribution, but the horseflies might also fly, for example, at 90° with respect to the sun, which would result in bimodal distribution data. To account for this possibility, we performed the MMR test on transformed data according to (Moore 1980; Dreyer et al. 2021). Briefly, the segment means were first multiplied by 2 with subsequent subtraction of 360° from all angles >360°. This transforms a bimodal circular distribution to unimodal, which can be analysed with MMR method using unmodified vector lengths as weights.

To analyse the potential observer/setup effect we transformed the angular data, by subtracting the estimated observer angles from the obtained means of all the animals recorded with the corresponding setup. The directional analysis was performed as above.

## Results

### Attraction preference

Tray colour for subsequent testing was chosen in a preliminary assay of horsefly preference, where horsefly visits to four differently coloured trays were observed for 1 h. The UV-reflective white tray had a low DOLP across all spectral bands, while the unpainted tray had a high DOLP in the UV. Both white trays did not receive any visits at all. The black and green trays with a high DOLP in the UV and blue range were expected to attract the horseflies, as horsefly polarotaxis is mediated by UV- and blue-sensitive photoreceptors. However, the green tray received <10 visits, while the black water tray proved to be most attractive with >100 visits, corroborating our previous observation that horsefly polarotaxis is triggered by dark objects reflecting polarised light (Meglič et al. 2019). For the recordings, the black tray was chosen. A few recordings were performed with a dark red tray, which has proven as effective as the black tray, but offered a slightly better contrast with the horseflies. Horseflies have vision limited to 300–600 nm range and probably perceive dark red (reflecting >600 nm) as black (Meglič et al. 2019).

### Description of the behaviour

The horsefly “drinking on the wing” behaviour can be separated into three phases: approach, water collection and departure (Mov. 1–3). In our analysis, the water collection phase included the descent towards the water surface, water touch-down/landing, and the animal’s take-off from the water surface. The horseflies spent more time approaching and inspecting the water tray than departing away from it (SI Figure 4). During approach, the horsefly generally flew a few times around and above the water tray (SI Figure 5). These inspections were done at much lower speeds compared to the conspecifics that were not interested in the water tray, making it easier for us to record the drinking events. In the following text, we report the medians that were calculated from averaged values per animal in different flight phases (for the 1st and 3rd quartiles see Table 1). The horseflies inspected the water tray at low altitudes and speeds (Fig. 3; Table 1). The median total approach speed (in 3D) was 1.57 m/s; the horizontal approach speed was 1.54 m/s and was higher than the vertical speed 0.17 m/s. During approach, the horseflies gradually reduced their altitude with a slight negative vertical velocity of −0.05 m/s, but only up to the start of the water collecting phase. This was characterised by a quick plunge onto the water surface which occurred around *t* = −135 ms before water contact (Fig. 3).

**Table 1:** Approach and departure flight phases differ in their horizontal and vertical speed, and altitude

	Approach	Departure	*p*-value	Descent and landing
Altitude (mm)	53.52 (40.12, 65.02)	70.6 (53.21, 91.65)	<0.01	18.37 (15.29, 21.35)
Total speed (m/s)	1.57 (1.32, 1.79)	1.50 (1.19, 1.75)	<0.01	0.6 (0.48, 0.73)
Horizontal speed (m/s)	1.54 (1.29, 1.77)	1.19 (0.94, 1.49)	<0.01	0.48 (0.32, 0.62)
Vertical speed (m/s)	0.17 (0.14, 0.24)	0.62 (0.38, 0.81)	<0.01	0.28 (0.22, 0.33)
Vertical velocity (m/s)	−0.05 (−0.11, 0.0)	0.62 (0.34, 0.81)	<0.01	−0.18 (−0.22, −0.13)

Medians and quartiles (*q*1, *q*3) calculated from entire paths (i.e. approach not limited to −1 s < *t* < *t*_descent start_)

During descent, the horseflies performed a combination of yaw, pitch and body roll rotations that changed their heading by approximately 180° (Fig. 2D; Mov. 1–3), reduced their speed (Fig. 3A, E) and positioned them with their heads pointing away from the water (positive pitch angles in Fig. 2D, SI Figures 2, 3). This resulted in the animals generally touching the water abdomen-first, followed by the legs and then with the rest of their body. The average water contact lasted approximately 73 ± 11 ms, which is a slight overestimation due to our definition of the water landing. After collecting the water droplet, the horseflies generally departed the water tray by flying away and out of the camera field of view in a straight line (SI Figure 5). The horseflies departed by flying at higher altitudes (70.6 mm) and lower total (1.50 m/s) and horizontal (1.19 m/s) speeds compared to the approach (Table 1). Their vertical speed was 0.62 m/s, which is higher than during approach. A few horseflies circled the water tray even after water landing/collection (SI Figure 5).

### Directional preferences

**Fig. 4:**
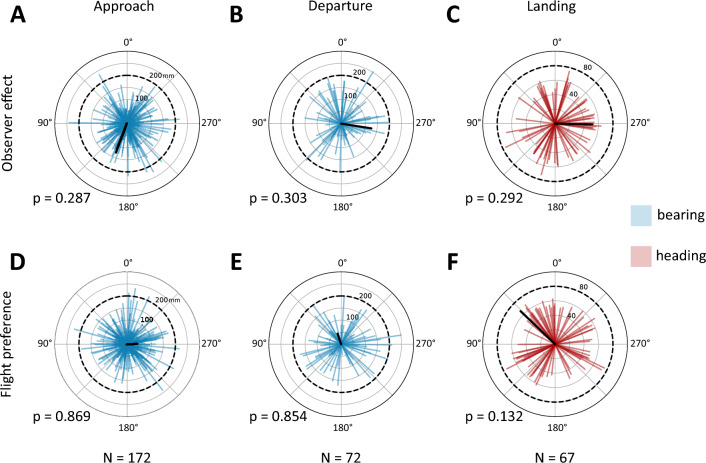
Horseflies showed no directional flight preferences. Polar plots of mean bearing (blue) and heading (red) direction of all analysed straight flight segments [approach (**A**, **D**), departure (**B**, **E**)] and water landing (**C**, **F**). The vector lengths indicate the animal’s “directedness” in corresponding segments. The black vectors’ magnitude represents the R* statistics of the Moore’s modified Rayleigh test, while their direction is the mean angle of the bearing/heading vectors in corresponding graphs. The dashed circles indicate the limit for statistical significance for R* (*p* ≤ 0.05). *P*-values are indicated in the bottom left of each plot. The observer (top row) or sun (bottom row) are located at 0°. The approach phase was generally longer (see SI Figure 4) than the departure phase, which resulted in a greater number of isolated segments (*N*). The flight was not affected by the position of the recording setup and the observer, nor by the location of the sun

The directional analysis was done on combined data obtained on different days and with different camera setups. We isolated more straight flight segments during approach than during departure, which reflects the length of time the animals spent inspecting the water tray [Fig. 4, (first two columns), SI Fig. 4]. We found no evidence that the camera setup or the observer had any effect on the animals’ approach behaviour (Fig. 4A–C). The MMR test *p*-values were well above the significance level: 0.287 (approach), 0.292 (landing) and 0.303 (departure). Similarly, the sun direction had no effect on the bearing nor heading and the horseflies showed no directional flight preferences in any of the flight phases (Fig. 4D–F). The MMR test *p*-values were: 0.869 (approach), 0.132 (landing) and 0.854 (departure).

## Discussion

In this study, we show that horsefly water detection and collection is at a short range (<1 m) a robust visually guided behaviour, independent of the position of celestial or terrestrial visual landmarks. The preferred low flight altitude implies that the horseflies attempt to view the water body with the broadest possible angular extent of photoreceptors (across the azimuth). Nevertheless, the small tray in a large field (1 Ha) was sufficient to attract numerous horseflies, otherwise flying at approximately up to 2 m above ground, implying that the HPL is indeed a salient cue (SI Figure 7). This corroborates the previous findings showing that the degree of linear polarisation (DOLP) is an important factor in water detection (Kriska et al. 2009; Egri et al. 2012). Generally, the attraction is proportional to the DOLP of an object (Kriska et al. 2009). However, a high DOLP does not always elicit polarotaxis. For example, the green tray in our observations had a high DOLP in blue and UV (Fig. 1), but failed to attract horseflies. This is presumably due to a weak luminance/brightness contrast between the tray and the background. Additionally, the green tray likely caused avoidance behaviour as green colour was shown to repel the host-seeking female horseflies (Meglič et al. 2019). The taxis towards water is, therefore, complex and depends on both the chromatic and achromatic characteristics of the water body, as well as the physiological state and stimulation threshold of the animal (Schwind 1991; Kriska et al. 2009).

The absence of any angular flight preference (Fig. 4) suggests that horsefly polarisation vision operates across a broad range of light intensities, ranging from very bright direct (solar) and much dimmer indirect (skylight, vegetation) reflections from the water surface. The lack of angular preference is likely due to the stochastic retinal architecture. In the horsefly eye, the high PS ommatidia housing receptors with high PS are distributed across their entire retina, although their density is greater ventrally (Meglič et al. 2019). This is in contrast to *Notonecta* with a highly specialised and regionalised compound eye that explains their alignment of the eye’s ventral retinal band with the Brewster’s angle during the water dive (Schwind 1983). The randomly spread PS ommatidia in the horseflies, therefore, allow the detection of various targets within a complex and changing environment and in various body orientations. One situation where the target detection seems to fail is when the prey animals hide in the shade (Horváth et al. 2010). This reduces the intensity of polarised light reflected from the fur, which makes the prey less or even not attractive to the horseflies (Horváth et al. 2010). We did not test the water collection behaviour in the shade and it would be interesting to determine whether the horseflies can detect water in such conditions.

Horsefly drinking behaviour encompasses a remarkable flight pattern, which leads to a deliberate touch-down: the period just before the plunge is denoted by a 180° body turn, a brief backwards-oriented flight and the observation of the water surface at a very low speed. The slow flight speed and the extended observation of the reflected light pattern cause a longer exposure and an increased signal to noise ratio in the PS photoreceptors, which likely facilitates water identification and the decision to plunge. While the turn serves to decelerate the animal, it also reverses the optic flow in the horizontal plane, thereby evoking a large change in the signalling pattern within the neural circuitry for motion detection that is likely associated with the initiation of the descent, but is possibly also suppressed by the efference copy (Hardcastle and Krapp 2016).

We assume that the horseflies drink the collected water during flight to mitigate the water loss accrued during the day. Insects lose most of the water though their ventilation system (spiracles) (Prange 1996). Activities requiring high oxygen input such as flight also increase the insect’s ventilation and therefore water loss (Weis-Fogh 1967; Nicolson and Louw 1982; Wegener 1996; Reinhold 1999; Niven and Scharlemann 2005). The horsefly thorax and abdomen are perforated with numerous spiracles (Bromley 1926), presumably due to the high oxygen demand of the flight muscles. We hypothesise that these spiracles are open during flight, leading to increased water loss due to ventilation, particularly during hot and dry conditions such as those experienced in the summer. Furthermore, the high temperatures may also lead to heat stress, which is mitigated either behaviourally or by evaporative cooling. The latter again increases the insect’s ventilation rate and evaporative water loss (Willmer 1982; Prange, Henry D. 1996). Whether horseflies use evaporative cooling or is the convective heat transfer during flight a sufficient cooling mechanism is unknown. Nevertheless, high water loss during flight in a hot environment is expected and it is likely that the horseflies seek the water for both drinking and cooling. Interestingly, certain dipterans engage in the so-called bubbling behaviour, when the animal continually regurgitates and reabsorbs a droplet of liquid. Although bubbling is most often associated with feeding, it is also used for cooling (Hendrichs et al. 1992; Gomes et al. 2018). If horseflies perform such actions during flight, the convective air flow around the droplet might further improve the cooling effect of bubbling. However, drinking on the wing may have other advantages. The question remains why horseflies evolved such “drinking on the wing” behaviour. Despite the high surface tension of water and the hydrophobic nature of the insect cuticle which reduce the chances of drowning, non-aquatic insects generally avoid plunging directly into the water. Drinking water can be easily done by landing and imbibing the water while grounded. Not only does this reduce the risk of drowning, but also it likely uses up less energy compared to drinking during flight with high energy demands (Wegener 1996; Reinhold 1999; Niven and Scharlemann 2005). The horsefly water collection strategy may offer certain advantages that outweigh the risk of drowning.

One advantage could be that landing poses a threat to the horsefly, which may be safer from predators while flying. Alternatively, “drinking on the wing” might be a time-saving mechanism as it allows the horsefly to multitask by simultaneously search for hosts and/or mates. This is particularly important during high water loss that would lead to long drinking times and, therefore, long “ground” time. Drinking on the wing might, therefore, be an adaptation for optimising mate/prey searching time in light of metabolic flight requirements by reducing the “ground time” to the short pitstop on the water surface. Remarkably, horseflies (*T. bromius* and a few larger *T. bovinus*) were almost the only insects performing the short water pitstops. On a few occasions, we observed an unidentified wasp performing a similar manoeuvre, but without the prominent 180° yaw turn, possibly because it was flying at a much slower speed.

To summarise, horsefly polarotaxis is a robust mechanism of water detection independent of the light intensity during daytime, but influenced by the chromatic and polarisation properties of the reflected light. By relying on the HPL, horseflies are capable of detecting a potential water puddle from far away (Horváth et al. 2008; Egri et al. 2012). However before collecting the water, a closer inspection of the target is required. This is done at low speeds and at altitudes coinciding with strongest HPL cues (SI Figure 7). We conclude that the horseflies rely on HPL for both long- and short-range water detection.

### Supplementary Information

Below is the link to the electronic supplementary material.

Supplementary file1 (PDF 2852 KB)

Supplementary file2 (MP4 3162 KB)

Supplementary file3 (MP4 73157 KB)

Supplementary file4 (MP4 52344 KB)
