# Supplementary material for: Drinking on the wing: water collection in polarotactic horseflies
Source: J Comp Physiol A Neuroethol Sens Neural Behav Physiol. 2023 Jul 21;209(6):943–54. doi: 10.1007/s00359-023-01657-3 (PMC10643286; doi:10.1007/s00359-023-01657-3)
Supplement: Supplementary file 1 — Supplementary file1 (PDF 2852 KB) [file 359_2023_1657_MOESM1_ESM.pdf]

Drinking on the wing: water collection in polarotactic horseflies

Uroš Cerkvenik, Gregor Belušič

Supplementary information

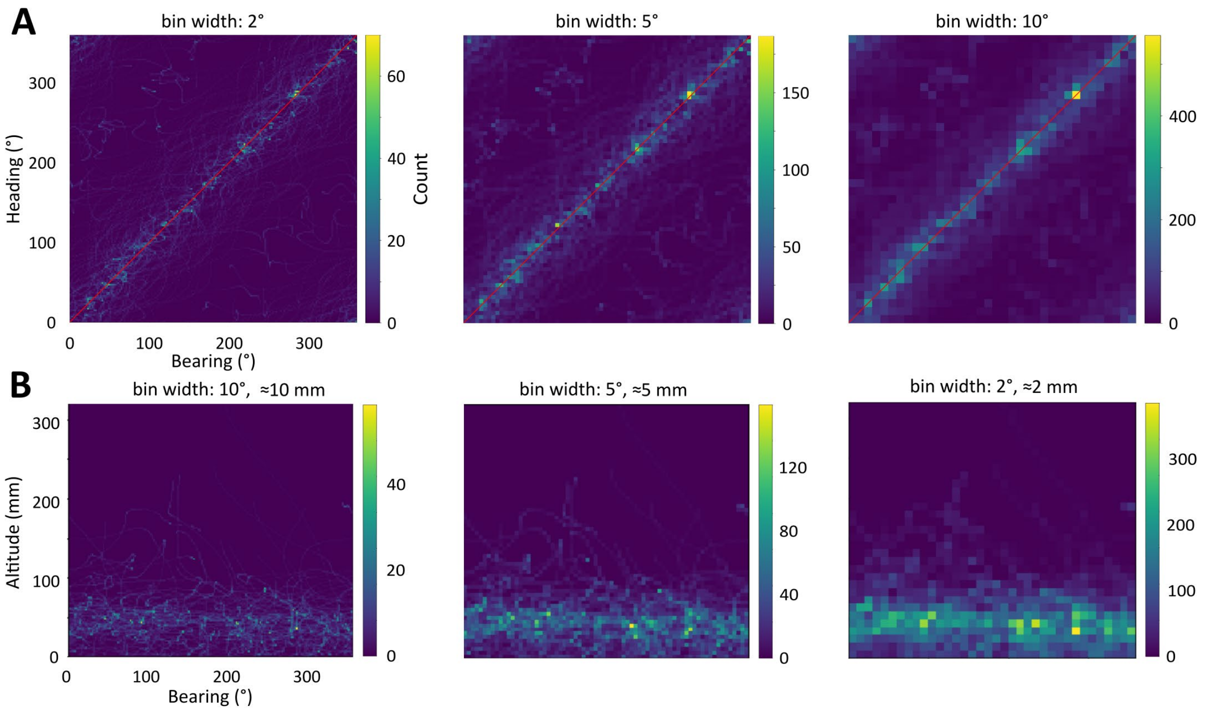

SI Fig. 1. Bearing strongly correlates with the heading direction, but not with altitude. A) Heatmaps showing the frequency of binned heading and bearing data for all analysed videos. The heading-bearing relationship closely follows the y = x line (red) and is not dependent on the bin size. **B)** The heatmaps of binned altitude and bearing show that flight direction does not depend on its altitude.

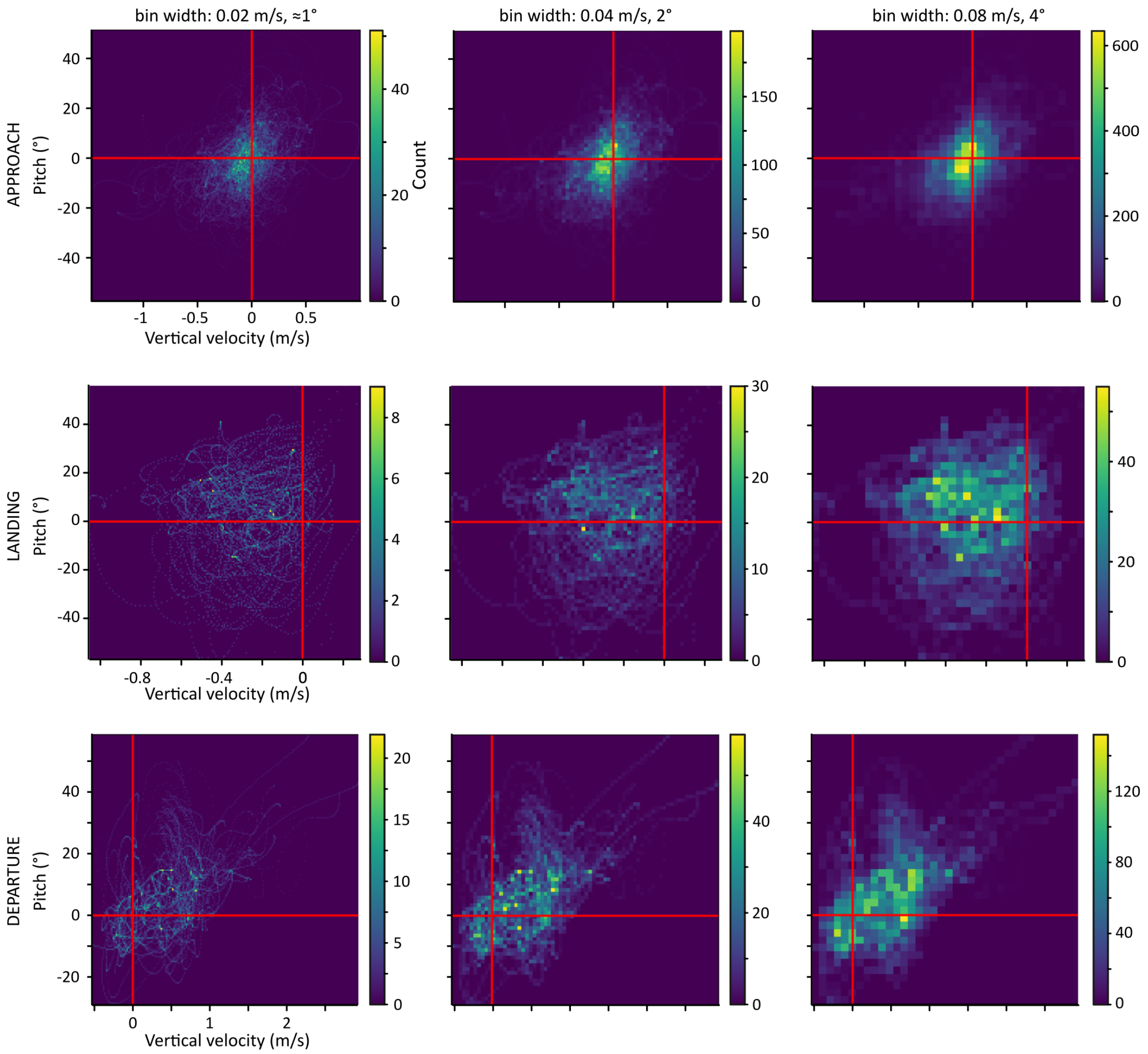

SI Fig. 2. Horseflies generally fly horizontally during approach; but pitch upwards during landing and departure. Heatmaps of binned pitch data plotted against vertical velocity for different flight phases. During approach (top row), the pitch oscillates equally around 0°. During landing on the water surface (middle row) and departure (bottom row), the animals pitch highly upward, indicated by the high density of datapoints in the 1st and 2nd quadrants. Data binning has no effect on this observation. Red lines indicate the origin of the coordinate system.

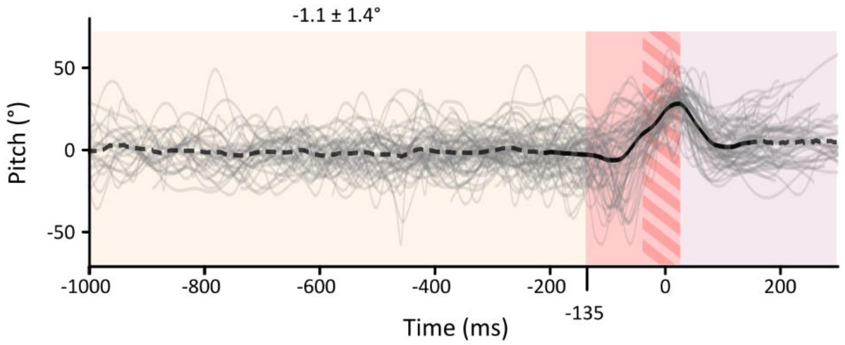

SI Fig. 3. Horseflies pitch upward upon landing on the water. In general, the pitch of flying horseflies oscillates around 0° (mean -1.1 ± 1.4°). This changes during descent and landing on the water surface, when the animals consistently pitch upwards, which results in them contacting the water surface with their abdomen first, followed by the legs, and the rest of the body (Mov. 1-3). Colour code same as in Fig. 3.

SI Table 1.**The horseflies had no preferred flight directions and were not influenced by the observers.** Comparison of Moore-Rayleigh statistical results (p-values) when assuming unimodal or bimodal distribution of bearing (approach/departure) or heading data (landings) across different segmentation parameters (r_κ). The number of segments used in the analysis is provided in brackets. This shows the robustness of the segmentation procedure as the number of straight flight segments differs little across three different curvature thresholds. Example of data can be seen in Fig. 4.

	**unimodal**			**bimodal**			
***r_κ threshold* (mm)**	**>105**	**>210**	**>320**	**>105**	**>210**	**>320**	
**approach**	0.982 (182)	0.869 (172)	0.875 (161)	0.155 (182)	0.260 (172)	0.615 (161)	*flight preference*
**landing** *(heading)*	0.132 (67)			0.674 (67)			*flight preference*
**departure**	0.846 (75)	0.854 (72)	0.827 (68)	0.592 (75)	0.445 (72)	0.409 (68)	*flight preference*
**approach**	0.418 (182)	0.287 (172)	0.129 (161)	0.089 (182)	0.160 (172)	0.107 (161)	*observer effect*
**landing** *(heading)*	0.292 (67)			0.355 (67)			*observer effect*
**departure**	0.349 (75)	0.303 (72)	0.319 (68)	0.622 (75)	0.447 (72)	0.359 (68)	*observer effect*

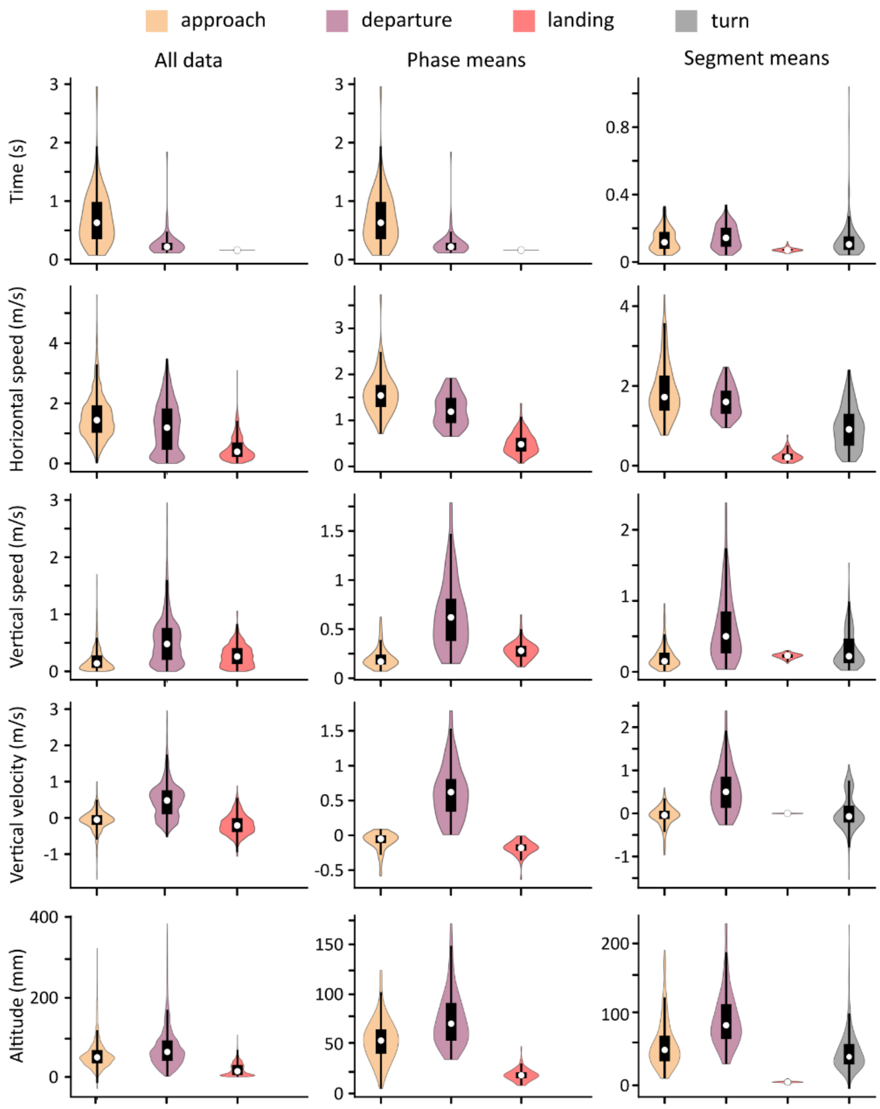

SI Fig. 4. Distributions of data in different flight phases. Left column shows distributions of all data that are highly skewed due to small number of outliers. Averaging (middle column) and segmentation of straight flights and turns (right column) reduce the skewness but retain the overall shape and characteristics of the distributions. Colours: different flight phases, white circles: median values, black box and whiskers plots: quartiles.

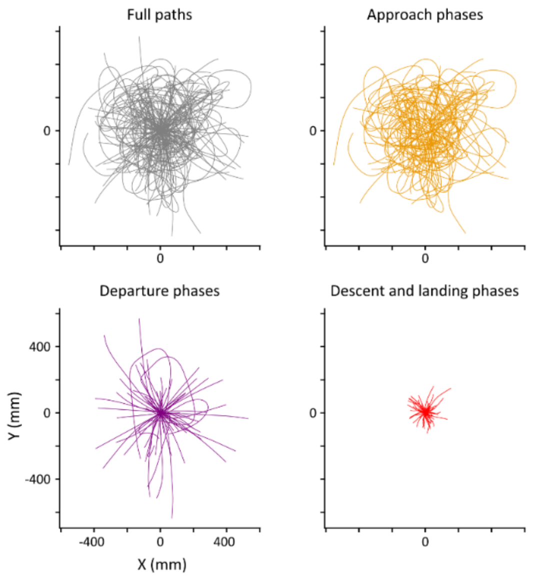

SI Fig. 5. 2D flight trajectories. Flight trajectories projected onto the XY plane. Grey: full paths; orange: approach phase (t < -135 ms); purple: departure phase (t > 29 ms); red: descent and landing phase (-1345 ms< t <29 ms). Sun direction is along the positive X axis.

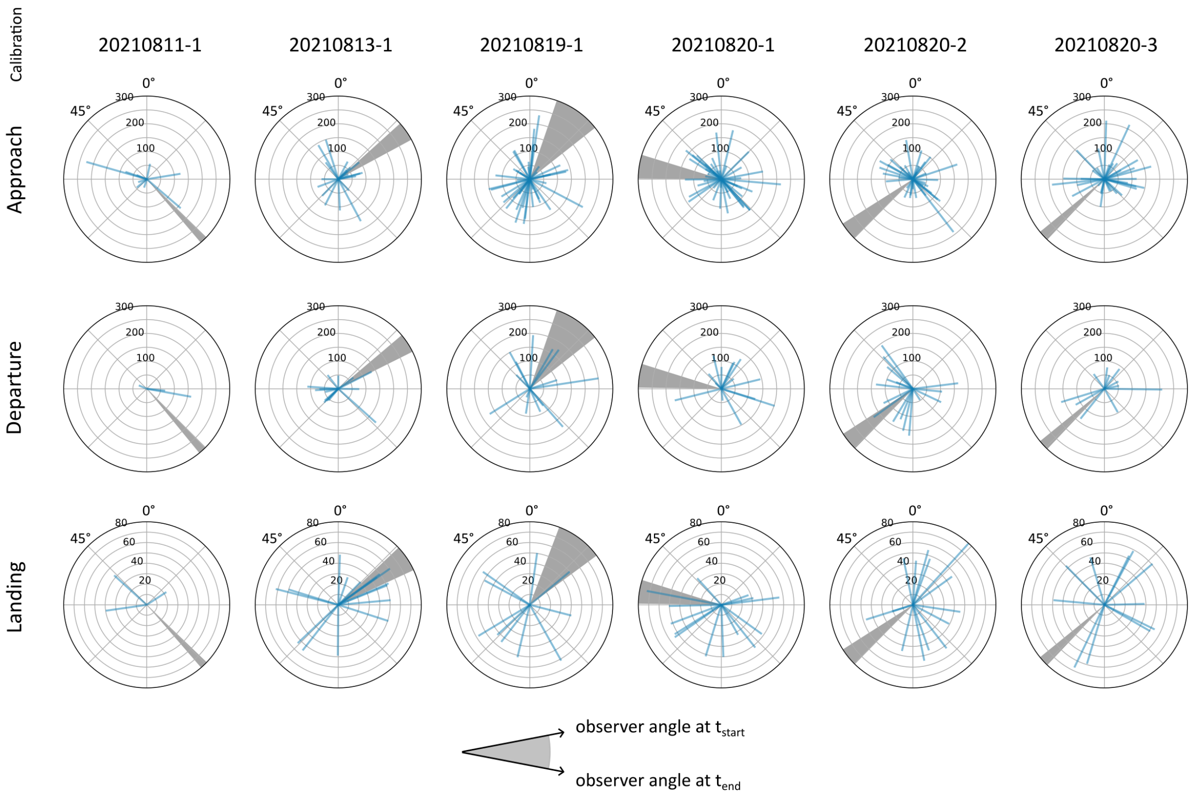

SI Fig. 6. Bearing and observer angles per calibration day or camera setup. The polar bar plots show mean bearing segment vectors during approach, departure and water landing phase. The observer angle is indicated with a dark polar bar and shows the range of observer angles during the whole observation period.

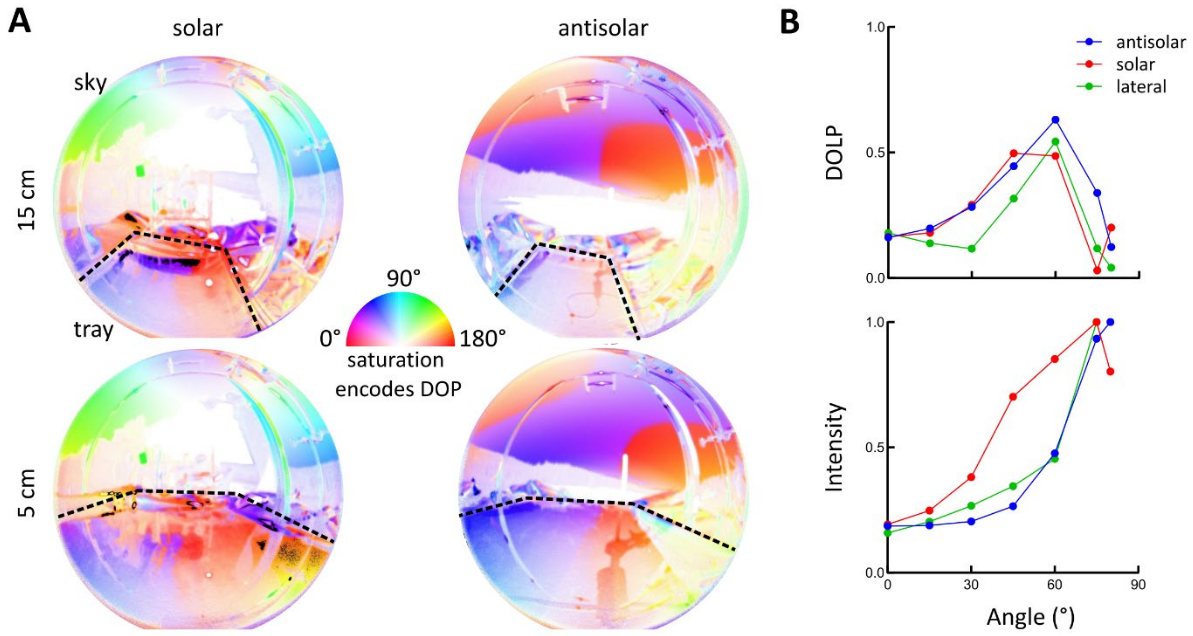

SI Fig. 7. Polarization cues of the water-filled trays. A) Degree and angle of polarized skylight and tray reflections at two different altitudes. The HPL cues are moderate at 15 cm above the surface and increase with decreasing altitude. The skylight is always strongly polarized but has a specific pattern of polarization ranging from vertical to horizontal polarization, whereas water reflections are always polarized horizontally. **B)** Intensity and degree of linear polarization (DOLP) of light reflected from water trays changes with the viewing angle and location of the sun. DOLP is highest at viewing angles close to the Brewster's angle (≈ 53° for air-water interface). Intensity of the direct sun reflections (solar) increases faster with the viewing angle compared to antisolar or lateral reflections.
